# Crystal structure of di­chlorido­[2-(di­phenyl­phosphan­yl)-3,4,5,6-tetra­fluoro­benzene-1-thiol­ato-κ^2^ *P*,*S*]gold(III)

**DOI:** 10.1107/S2056989015016758

**Published:** 2015-09-17

**Authors:** Peter W. R. Corfield, Mary Bailey

**Affiliations:** a Department of Chemistry, Fordham University, 441 East Fordham Road, Bronx, NY 10458, USA; b Department of Chemistry, The Ohio State University, Columbus, Ohio 43210, USA

**Keywords:** crystal structure, mixed ligand, gold complex

## Abstract

The title compound, [Au(C_18_H_10_F_4_PS)Cl_2_], crystallizes as neutral mol­ecules, with the Au^III^ atom coordinated by two Cl atoms and by the P and S atoms of the bidentate phosphanyl thiol­ate ligand, in a slightly distorted square-planar environment. The mol­ecules are linked into centrosymmetric dimers *via* long axial Au—Cl bonds of 3.393 (4) Å. This axial Au—Cl distance is longer than is usually seen, although one other example has been given. Dimer formation may explain the unexpectedly low solubility of the compound in common polar solvents. There is also a separate inter­molecular Au—F contact of 3.561 (6) Å, but this distance seems too long to be regarded as a bond. Two putative C—H⋯F hydrogen bonds appear to link the dimers into sheets parallel to (110). There is a short inter­molecular F⋯F contact of 2.695 (10) Å between two dimers related by the twofold axis.

## Related literature   

For synthetic details, see: Eller (1971 ▸); Eller & Meek (1970 ▸). Hollis & Lippard (1983 ▸) describe a similarly long axial Au—Cl bond in a mixed-valence gold compound, although other axial Au—Cl bonds in the literature are in the 3.0–3.1 Å range, as in Elder & Watkins (1986 ▸).
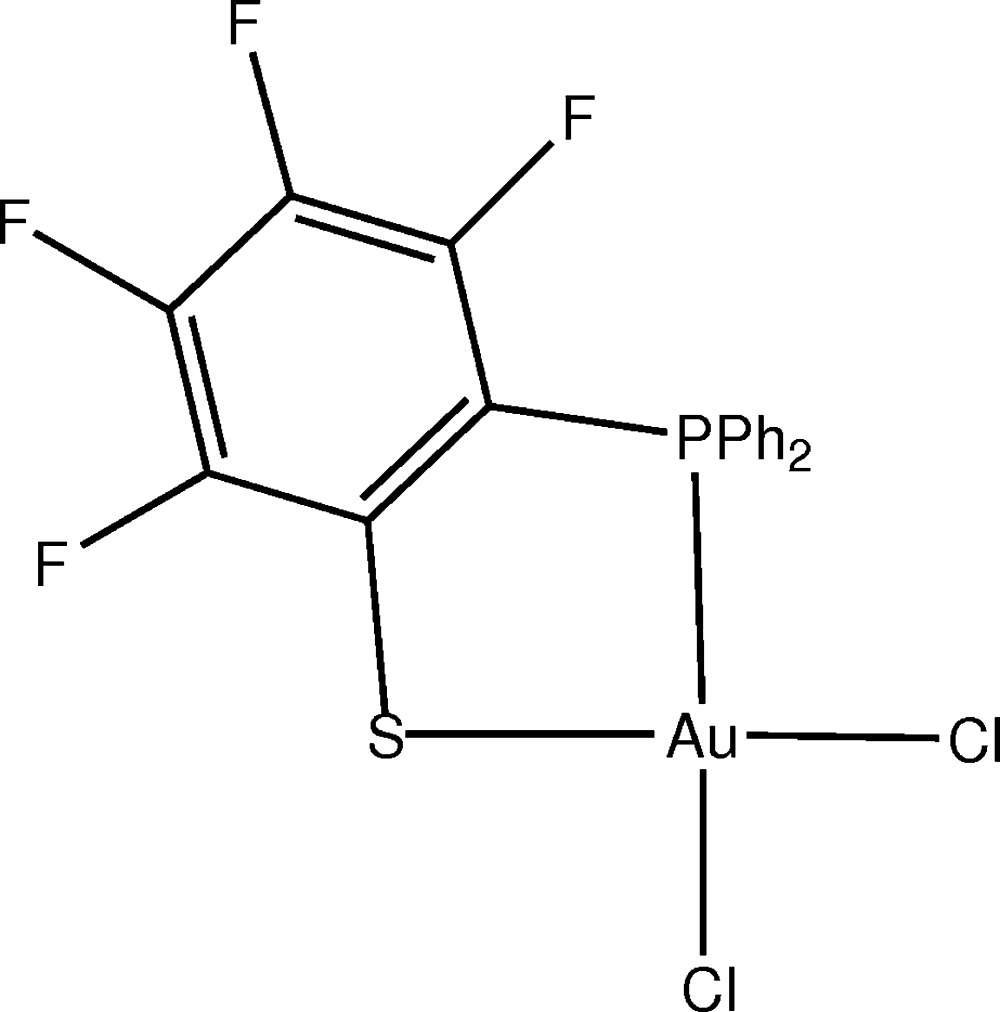


## Experimental   

### Crystal data  

[Au(C_18_H_10_F_4_PS)Cl_2_]
*M* *_r_* = 633.16
Monoclinic, 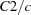

*a* = 18.90 (2) Å
*b* = 8.388 (12) Å
*c* = 24.15 (3) Å
β = 100.75 (3)°
*V* = 3761 (8) Å^3^
*Z* = 8
Mo *K*α radiation
μ = 8.15 mm^−1^
*T* = 298 K
0.22 × 0.16 × 0.13 mm

### Data collection  

Picker 4-circle diffractometer
Absorption correction: gaussian (Busing & Levy, 1957 ▸) *T*_min_ = 0.384, *T*_max_ = 0.442
4262 measured reflections
4142 independent reflections
3209 reflections with *I* > 2σ(*I*)
*R*_int_ = 0.025
18 standard reflections every 500 reflections intensity decay: −1.0(3)

### Refinement  

*R*[*F* ^2^ > 2σ(*F* ^2^)] = 0.032
*wR*(*F* ^2^) = 0.086
*S* = 1.03
4142 reflections
220 parameters
H-atom parameters constrained
Δρ_max_ = 1.00 e Å^−3^
Δρ_min_ = −0.87 e Å^−3^

### 

Data collection: Corfield (1972 ▸); cell refinement: Corfield (1972 ▸); data reduction: Corfield *et al.* (1973 ▸); program(s) used to solve structure: Corfield (1972 ▸); program(s) used to refine structure: *SHELXL2014* (Sheldrick, 2015 ▸); molecular graphics: *ORTEPIII* (Burnett & Johnson, 1996 ▸); software used to prepare material for publication: *SHELXL2014*.

## Supplementary Material

Crystal structure: contains datablock(s) I. DOI: 10.1107/S2056989015016758/wm5209sup1.cif

Structure factors: contains datablock(s) I. DOI: 10.1107/S2056989015016758/wm5209Isup2.hkl

**Fig. 1.**
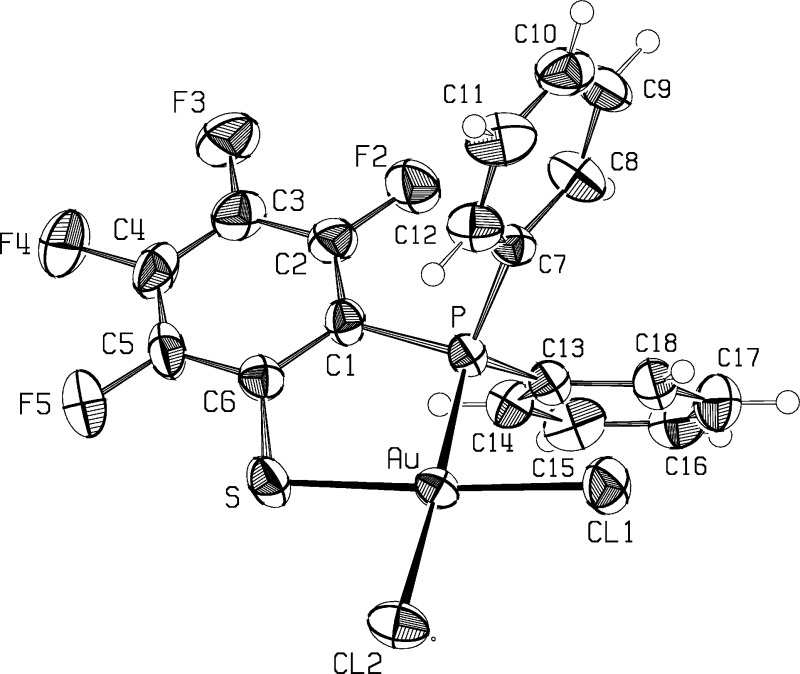
The molecular structure of the title compound, with displacement ellipsoids drawn at the 50% probability level.

**Fig. 2.**
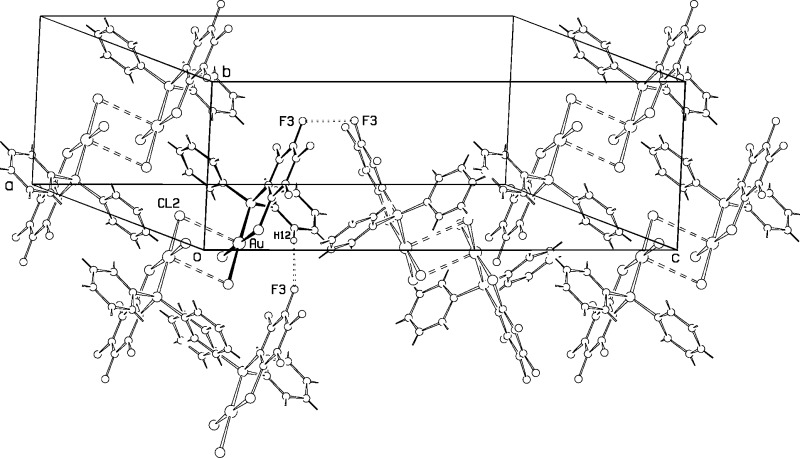
Packing of the title complex, viewed along a direction near to the a axis. The centrosymmetric dimers are shown, as well as the proximity of F3(1,y - 1,z) to a sixth coordination site for the gold atom. Long Au—Cl bonds are gived as dashed lines.

. DOI: 10.1107/S2056989015016758/wm5209fig1.tif
The mol­ecular structure of the title compound, with displacement ellipsoids drawn at the 50% probability level.

a y z . DOI: 10.1107/S2056989015016758/wm5209fig2.tif
Packing of the title complex, viewed along a direction near to the *a* axis. The centrosymmetric dimers are shown, as well as the proximity of F3(1,*y* − 1,*z*) to a sixth coordination site for the gold atom. Long Au—Cl bonds are gived as dashed lines.

CCDC reference: 1422931

Additional supporting information:  crystallographic information; 3D view; checkCIF report

## Figures and Tables

**Table:** 

AuS	2.273(3)
AuP	2.258(3)
AuCl2	2.337(3)
AuCl1	2.305(3)

**Table:** 

SAuP	90.22(10)
SAuCl2	87.51(10)
PAuCl2	177.69(6)
SAuCl1	176.59(7)
PAuCl1	88.36(10)
Cl2AuCl1	93.88(11)
SAuCl2^i^	88.12(7)
PAuCl2^i^	90.45(9)
Cl2AuCl2^i^	89.91(9)
Cl1AuCl2^i^	94.99(7)

Symmetry code: (i) 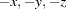.

**Table 2:** Hydrogen-bond geometry (, )

*D*H*A*	*D*H	H*A*	*D* *A*	*D*H*A*
C12H12F3^ii^	0.93	2.60	3.444(7)	151
C18H18F4^iii^	0.93	2.50	3.082(7)	121

Symmetry codes: (ii) 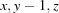; (iii) 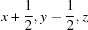.

## supplementary crystallographic information

### S1. Synthesis and crystallization

The preparation of the compound is described by Eller (1971), and synthesis of the then novel ligand is given in Eller & Meek (1970).

### S2. Refinement

To reduce the number of parameters varied, the phenyl groups C16—C21 and C22—C27 were constrained as rigid hexagons, with C—C distances of 1.385 Å. Aromatic H atoms were placed geometrically, with their *U*_eq_ values set 1.2 times the *U*_iso_ of their bonded C atoms.

### Crystal data

**Table:**

[Au(C_18_H_10_F_4_PS)Cl_2_]	*F*(000) = 2384
*M**_r_* = 633.16	*D*_x_ = 2.236 Mg m^−^^3^*D*_m_ = 2.181 (3) Mg m^−^^3^*D*_m_ measured by flotation in carbon tetrachloride/bromoforom mixture. Discrepancy may be due to an uncalibrated pycnometer.
Monoclinic, *C*2/*c*	Mo *K*α radiation, λ = 0.7107 Å
*a* = 18.90 (2) Å	Cell parameters from 24 reflections
*b* = 8.388 (12) Å	θ = 4.2–25.1°
*c* = 24.15 (3) Å	µ = 8.15 mm^−^^1^
β = 100.75 (3)°	*T* = 298 K
*V* = 3761 (8) Å^3^	Irregular, red
*Z* = 8	0.22 × 0.16 × 0.13 mm

### Data collection

**Table:**

Picker 4-circle diffractometer	3209 reflections with *I* > 2σ(*I*)
Radiation source: sealed X-ray tube	*R*_int_ = 0.025
Oriented graphite 200 reflection monochromator	θ_max_ = 27.5°, θ_min_ = 2.5°
θ/2θ scans	*h* = 0→24
Absorption correction: gaussian (Busing & Levy, 1957)	*k* = 0→9
*T*_min_ = 0.384, *T*_max_ = 0.442	*l* = −31→30
4262 measured reflections	18 standard reflections every 500 reflections
4142 independent reflections	intensity decay: −1.0 (3)

### Refinement

**Table:**

Refinement on *F*^2^	Primary atom site location: heavy-atom method
Least-squares matrix: full	Secondary atom site location: difference Fourier map
*R*[*F*^2^ > 2σ(*F*^2^)] = 0.032	Hydrogen site location: inferred from neighbouring sites
*wR*(*F*^2^) = 0.086	H-atom parameters constrained
*S* = 1.02	*w* = 1/[σ^2^(*F*_o_^2^)] where *P* = (*F*_o_^2^ + 2*F*_c_^2^)/3
4142 reflections	(Δ/σ)_max_ < 0.001
220 parameters	Δρ_max_ = 1.00 e Å^−^^3^
0 restraints	Δρ_min_ = −0.87 e Å^−^^3^

### Special details

**Table:**

Experimental. Data reduction followed procedures in Corfield *et al.* (1973), with programs written by Corfield and by Graeme Gainsford.
Geometry. All e.s.d.'s (except the e.s.d. in the dihedral angle between two l.s. planes) are estimated using the full covariance matrix. The cell e.s.d.'s are taken into account individually in the estimation of e.s.d.'s in distances, angles and torsion angles; correlations between e.s.d.'s in cell parameters are only used when they are defined by crystal symmetry. An approximate (isotropic) treatment of cell e.s.d.'s is used for estimating e.s.d.'s involving l.s. planes.
Refinement. To reduce the number of parameters varied, the phenyl groups C16—C21 and C22—C27 were constrained as rigid hexagons, with C—C distances of 1.385 Å. *U*_eq_ values for the aromatic H atoms were set 1.2 times the *U*_iso_ of their bonded C atoms.

### Fractional atomic coordinates and isotropic or equivalent isotropic displacement parameters (Å^2^)

**Table:**

	*x*	*y*	*z*	*U*_iso_*/*U*_eq_	
Au	0.03240 (2)	0.02779 (3)	0.08580 (2)	0.03238 (9)	
Cl1	0.14260 (10)	−0.0955 (2)	0.09200 (9)	0.0544 (5)	
Cl2	−0.03221 (10)	−0.1909 (2)	0.04280 (8)	0.0503 (4)	
S	−0.07524 (9)	0.1492 (2)	0.08537 (8)	0.0438 (4)	
P	0.09063 (8)	0.2433 (2)	0.12802 (7)	0.0309 (3)	
F2	0.1031 (2)	0.5694 (5)	0.1812 (2)	0.0572 (12)	
F3	−0.0040 (3)	0.7682 (6)	0.1937 (2)	0.0681 (13)	
F4	−0.1413 (3)	0.6750 (6)	0.1616 (2)	0.0757 (15)	
F5	−0.1734 (2)	0.3959 (6)	0.1090 (2)	0.0622 (12)	
C1	0.0205 (3)	0.3780 (8)	0.1371 (3)	0.0343 (14)	
C2	0.0351 (4)	0.5243 (9)	0.1633 (3)	0.0431 (16)	
C3	−0.0190 (4)	0.6241 (9)	0.1708 (3)	0.0479 (18)	
C4	−0.0886 (4)	0.5795 (9)	0.1527 (3)	0.0488 (18)	
C5	−0.1042 (3)	0.4341 (10)	0.1269 (3)	0.0461 (18)	
C6	−0.0507 (3)	0.3312 (8)	0.1186 (3)	0.0363 (14)	
C7	0.1393 (2)	0.1964 (5)	0.19702 (13)	0.0336 (14)	
C8	0.2018 (2)	0.2774 (5)	0.21989 (18)	0.0490 (18)	
H8	0.2215	0.3507	0.1981	0.059*	
C9	0.2350 (2)	0.2494 (6)	0.27517 (19)	0.058 (2)	
H9	0.2769	0.3038	0.2905	0.070*	
C10	0.2057 (3)	0.1403 (6)	0.30757 (14)	0.055 (2)	
H10	0.2279	0.1215	0.3447	0.066*	
C11	0.1432 (3)	0.0592 (6)	0.28470 (17)	0.060 (2)	
H11	0.1235	−0.0140	0.3065	0.072*	
C12	0.1100 (2)	0.0873 (5)	0.22942 (18)	0.0462 (17)	
H12	0.0681	0.0329	0.2141	0.055*	
C13	0.1473 (2)	0.3410 (5)	0.08744 (17)	0.0352 (14)	
C14	0.12320 (19)	0.4760 (5)	0.05659 (18)	0.0400 (15)	
H14	0.0771	0.5146	0.0566	0.048*	
C15	0.1678 (3)	0.5534 (4)	0.02581 (18)	0.0487 (18)	
H15	0.1516	0.6441	0.0051	0.058*	
C16	0.2365 (2)	0.4959 (6)	0.0259 (2)	0.051 (2)	
H16	0.2664	0.5480	0.0052	0.062*	
C17	0.26061 (18)	0.3610 (6)	0.0567 (2)	0.0527 (19)	
H17	0.3068	0.3224	0.0568	0.063*	
C18	0.2160 (2)	0.2835 (5)	0.08750 (19)	0.0433 (16)	
H18	0.2323	0.1929	0.1082	0.052*	

### Atomic displacement parameters (Å^2^)

**Table:**

	*U*^11^	*U*^22^	*U*^33^	*U*^12^	*U*^13^	*U*^23^
Au	0.03286 (13)	0.03025 (15)	0.03249 (12)	−0.00327 (11)	0.00210 (8)	−0.00023 (11)
Cl1	0.0446 (10)	0.0441 (11)	0.0717 (13)	0.0115 (8)	0.0038 (9)	−0.0094 (9)
Cl2	0.0572 (11)	0.0408 (10)	0.0499 (10)	−0.0166 (8)	0.0022 (8)	−0.0058 (8)
S	0.0279 (8)	0.0472 (11)	0.0541 (10)	−0.0065 (7)	0.0017 (7)	−0.0028 (8)
P	0.0258 (7)	0.0295 (9)	0.0361 (8)	−0.0005 (6)	0.0022 (6)	0.0001 (6)
F2	0.048 (2)	0.046 (3)	0.074 (3)	−0.006 (2)	0.000 (2)	−0.019 (2)
F3	0.082 (3)	0.051 (3)	0.071 (3)	0.010 (3)	0.012 (3)	−0.023 (2)
F4	0.066 (3)	0.073 (4)	0.088 (4)	0.037 (3)	0.015 (3)	−0.013 (3)
F5	0.034 (2)	0.072 (3)	0.080 (3)	0.009 (2)	0.008 (2)	0.005 (3)
C1	0.030 (3)	0.031 (4)	0.041 (3)	0.004 (3)	0.005 (3)	0.001 (3)
C2	0.047 (4)	0.038 (4)	0.043 (4)	0.005 (3)	0.004 (3)	−0.008 (3)
C3	0.060 (5)	0.034 (4)	0.049 (4)	0.013 (3)	0.007 (4)	−0.003 (3)
C4	0.051 (4)	0.045 (5)	0.052 (4)	0.021 (4)	0.015 (3)	0.002 (3)
C5	0.025 (3)	0.065 (5)	0.048 (4)	0.010 (3)	0.005 (3)	0.013 (4)
C6	0.036 (3)	0.041 (4)	0.032 (3)	0.003 (3)	0.005 (3)	0.007 (3)
C7	0.037 (3)	0.031 (4)	0.031 (3)	0.004 (3)	0.001 (3)	0.001 (3)
C8	0.047 (4)	0.048 (5)	0.046 (4)	−0.011 (3)	−0.006 (3)	0.006 (3)
C9	0.050 (4)	0.065 (6)	0.051 (4)	−0.001 (4)	−0.012 (4)	−0.011 (4)
C10	0.068 (5)	0.054 (5)	0.040 (4)	0.016 (4)	0.001 (4)	−0.002 (4)
C11	0.089 (6)	0.053 (5)	0.039 (4)	−0.003 (5)	0.014 (4)	0.012 (3)
C12	0.054 (4)	0.040 (4)	0.044 (4)	−0.008 (3)	0.008 (3)	0.000 (3)
C13	0.031 (3)	0.036 (4)	0.038 (3)	0.000 (3)	0.003 (3)	0.000 (3)
C14	0.042 (4)	0.039 (4)	0.040 (3)	−0.001 (3)	0.009 (3)	0.002 (3)
C15	0.070 (5)	0.031 (4)	0.047 (4)	−0.007 (4)	0.017 (4)	0.004 (3)
C16	0.059 (5)	0.051 (5)	0.047 (4)	−0.017 (4)	0.017 (4)	−0.003 (3)
C17	0.041 (4)	0.062 (5)	0.057 (5)	−0.006 (4)	0.016 (3)	−0.002 (4)
C18	0.034 (3)	0.045 (4)	0.051 (4)	0.001 (3)	0.006 (3)	−0.001 (3)

### Geometric parameters (Å, º)

**Table:**

Au—S	2.273 (3)	C7—C12	1.3850
Au—P	2.258 (3)	C8—C9	1.3850
Au—Cl2	2.337 (3)	C8—H8	0.9300
Au—Cl1	2.305 (3)	C9—C10	1.3850
Au—Cl2^i^	3.393 (4)	C9—H9	0.9300
Au—F3^ii^	3.560 (6)	C10—C11	1.3850
S—C6	1.747 (7)	C10—H10	0.9300
P—C1	1.787 (6)	C11—C12	1.3850
P—C7	1.791 (4)	C11—H11	0.9300
P—C13	1.781 (4)	C12—H12	0.9300
F2—C2	1.331 (8)	C13—C14	1.3850
F3—C3	1.337 (9)	C13—C18	1.3850
F4—C4	1.328 (8)	C14—C15	1.3850
F5—C5	1.338 (8)	C14—H14	0.9300
C1—C6	1.393 (8)	C15—C16	1.3850
C1—C2	1.385 (10)	C15—H15	0.9300
C2—C3	1.360 (10)	C16—C17	1.3850
C3—C4	1.358 (10)	C16—H16	0.9300
C4—C5	1.375 (11)	C17—C18	1.3850
C5—C6	1.372 (9)	C17—H17	0.9300
C7—C8	1.3850	C18—H18	0.9300
			
S—Au—P	90.22 (10)	C5—C6—S	118.5 (5)
S—Au—Cl2	87.51 (10)	C1—C6—S	123.4 (5)
P—Au—Cl2	177.69 (6)	C8—C7—C12	120.0
S—Au—Cl1	176.59 (7)	C8—C7—P	121.0 (3)
P—Au—Cl1	88.36 (10)	C12—C7—P	118.7 (3)
Cl2—Au—Cl1	93.88 (11)	C7—C8—C9	120.0
S—Au—Cl2^i^	88.12 (7)	C7—C8—H8	120.0
P—Au—Cl2^i^	90.45 (9)	C9—C8—H8	120.0
Cl2—Au—Cl2^i^	89.91 (9)	C10—C9—C8	120.0
Cl1—Au—Cl2^i^	94.99 (7)	C10—C9—H9	120.0
S—Au—F3^ii^	88.98 (11)	C8—C9—H9	120.0
P—Au—F3^ii^	107.67 (12)	C11—C10—C9	120.0
Cl2—Au—F3^ii^	71.87 (12)	C11—C10—H10	120.0
Cl1—Au—F3^ii^	88.50 (11)	C9—C10—H10	120.0
Cl2^i^—Au—F3^ii^	161.66 (8)	C10—C11—C12	120.0
C6—S—Au	103.2 (2)	C10—C11—H11	120.0
C1—P—C7	106.8 (3)	C12—C11—H11	120.0
C1—P—C13	108.2 (3)	C11—C12—C7	120.0
C7—P—C13	110.8 (2)	C11—C12—H12	120.0
C1—P—Au	104.6 (2)	C7—C12—H12	120.0
C7—P—Au	111.53 (17)	C14—C13—C18	120.0
C13—P—Au	114.36 (18)	C14—C13—P	120.0 (2)
C6—C1—C2	119.7 (6)	C18—C13—P	120.0 (2)
C6—C1—P	118.5 (5)	C15—C14—C13	120.0
C2—C1—P	121.8 (5)	C15—C14—H14	120.0
F2—C2—C3	119.1 (6)	C13—C14—H14	120.0
F2—C2—C1	119.9 (6)	C16—C15—C14	120.0
C3—C2—C1	121.0 (7)	C16—C15—H15	120.0
C4—C3—C2	119.7 (7)	C14—C15—H15	120.0
C4—C3—F3	120.0 (7)	C17—C16—C15	120.0
C2—C3—F3	120.3 (7)	C17—C16—H16	120.0
F4—C4—C3	119.5 (7)	C15—C16—H16	120.0
F4—C4—C5	120.2 (7)	C16—C17—C18	120.0
C3—C4—C5	120.2 (6)	C16—C17—H17	120.0
C6—C5—F5	120.3 (7)	C18—C17—H17	120.0
C6—C5—C4	121.4 (6)	C17—C18—C13	120.0
F5—C5—C4	118.3 (6)	C17—C18—H18	120.0
C5—C6—C1	118.0 (6)	C13—C18—H18	120.0

Symmetry codes: (i) −*x*, −*y*, −*z*; (ii) *x*, *y*−1, *z*.

### Hydrogen-bond geometry (Å, º)

**Table:**

*D*—H···*A*	*D*—H	H···*A*	*D*···*A*	*D*—H···*A*
C12—H12···F3^ii^	0.93	2.60	3.444 (7)	151
C18—H18···F4^iii^	0.93	2.50	3.082 (7)	121

Symmetry codes: (ii) *x*, *y*−1, *z*; (iii) *x*+1/2, *y*−1/2, *z*.
